# Work-Family Conflict and Unethical Pro-family Behavior: The Mediating Effect of Threat Appraisal and the Moderating Effect of Family Collectivism Orientation

**DOI:** 10.3389/fpsyg.2022.951904

**Published:** 2022-07-18

**Authors:** Mozhi Li, Lanxia Zhang, Zhuo Zhang, Xin Hai

**Affiliations:** ^1^School of Business Administration, Northeastern University, Shenyang, China; ^2^School of Economics and Management, Ningxia Institute of Science and Technology, Shizuishan, China

**Keywords:** cognitive appraisal, unethical pro-family behavior, work-family conflicts, threat appraisal, family collectivism orientation

## Abstract

Unethical pro-family behavior (UPFB) is prevalent in organizations and has adverse effects on organizations, but very few studies have examined the factors that lead to UPFB. We use a cognitive appraisal theoretical framework to argue that employees’ unethical pro-family (UPFB) behavior results from work and family conflicts (WFC/FWC) are mediated by threat appraisal and moderated family collectivism orientation. Based on the questionnaire data of 496 full-time employees from two-time points, we found that WFC/FWC was positively correlated with UPFB where threat appraisal played a mediating role in this relationship; Family collectivism orientation strengthens the threat appraisal-UPFB relationship and the mediation relationship between WFC/FWC and UPFB *via* threat appraisal. These findings offer an understanding of the theoretical and practical implications which could help organizations reduce UPFB. Finally, we discuss possible directions for future research.

## Introduction

Work-family conflict refers to a role conflict caused by the limitation of time and energy, unable to play the dual roles of work and family at the same time, including work-family conflict due to excessive work tasks leading to lack of family responsibilities, and family. Overburdened family work conflict resulting in unfulfilled job roles. The importance of the family is self-evident. Efforts and contributions directed to the family are highly respected. The individual will tend to pursue the wellbeing of family or family members through legitimate resources or proceeds. However, it is common to abuse organizational resources available through work for family needs (Liu et al., 2020; Cheng K. et al., 2021). Liu firstly described such negative behavior as “unethical pro-family behavior” (UPFB), specifically, which means that an employee intentionally violates laws, norms, moral rules of an organization for the purpose of benefiting one’s family. There are three boundary conditions for this concept: First, pro-family immorality must be intentional, that is, committed consciously by members, not due to mistakes, mistakes, and unconsciousness. Secondly, it is starting point-oriented rather than result-oriented, that is, as long as immoral behavior is done for the benefit of the family, no matter what the result is, it is a pro-family immoral behavior. Finally, pro-family unethical behavior is based on altruistic motives, that is, an employee engages in an action that benefits the family, but is not pro-family unethical if its original purpose is self-interest (Liu et al., 2020). UPFB may include, but is not limited to, illegally securing a job for a family member, bringing company belongings home to meet one’s family’s needs, using work resources to handle family matters, and other unethical behaviors intended to benefit one’s family. How to effectively prevent and control the unethical behavior of employees in the workplace is an important issue of common concern in both academic and practical circles. Many UPFB actions involve unauthorized use of workplace resources (Liu et al., 2020). Research has documented the associations between UPFB and unfavorable work outcomes, such as the fomentation of a sense of unfairness, corruption (Liu et al., 2020), decreased job satisfaction (Arasli and Tumer, 2008), and impaired future development of the organization (Kaptein, 2008). Therefore, restraining employees’ UPFB has a significant effect on the beneficial development of an organization. According to resource conservation theory, work-family conflict may arise from the lack of resources at work. When employees invest inherent resources at work but are not supplemented by organizational resources, they will have negative consequences such as stress due to the threat of resource depletion, and the resulting negative emotions will migrate to non-work areas (such as family), affecting the behavior of individuals in the family, leading to conflicts between work and family, causing work-family conflict.

Regrettably, researchers have largely ignored what makes employees engage in UFPB. Very few studies have examined the factors that lead to UPFB (Liu et al., 2020; Cheng K. et al., 2021). Cheng suggested that weaken the inducers of UPFB and strengthen the inhibitors of UPFB were two ways to inhibit UPFB (Cheng K. et al., 2021). Given that employees with family financial pressures and experience workplace bullying were more possible to adopt moral disengagement strategies to make the moral self-regulation failure, they would exhibit more UPFB than other employees. When some enterprise employees face failures or even other serious social impacts due to their UPFBs, people gradually realize the seriousness of the consequences of pro-family unethical behaviors (Liu et al., 2020; Yao et al., 2021). Consequently, family financial pressures and experience workplace bullying represent examples of a way to weaken the inducers of UPFB. To some extent, family supportive behavior from a supervisor could inhibit employees’ UPFB (Cheng K. et al., 2021). Therefore, family supportive supervisor behavior would represent an example of a way to strengthen the inhibitors of UPFB (Cheng K. et al., 2021). In a word, existing research is insufficient to adequately explain the antecedents of UPFB. Therefore, this study builds on emerging research that focuses on the antecedents of UPFB. Although Liu noted that work-family conflict and family work conflict were likely connected with UPFB (Liu et al., 2020), the process by which UPFB was initially triggered by work-family conflict and/or family work conflict has not been clarified. A theoretical framework, which serves to explain how and under what conditions employees’ WFC/FWC result in UPFB, has yet to be developed. From the perspective of personal factors, the existing literature fails to provide a unified explanation, which psychological factors induce the unethical behavior of the family; from the perspective of situational factors, the existing literature has not yet explained the leadership thinking mode and the inability of the family to lead the family. Whether ethical behavior is a significant antecedent of employee pro-organizational unethical behavior.

To better understand why and how employees’ WFC/FWC result in UPFB, this study is based on cognitive appraisal theory. Cognitive appraisal theory has been particularly suitable for explaining the mechanism of the relationship between the perception of stress and subsequent coping processes in situations of work and family conflicts, considering that the nature of such conflicts as the stressor is chronic rather than acute (Sagy, 2002; Miao and Wang, 2017). Cognitive appraisal theory posits that stress elicits a person’s primary appraisal (evaluate the value of situations) and secondary appraisal (determine the way to cope with adverse situations) (Lazarus and Folkman, 1984). We argue that WFC/FWC as a stressor elicits threat appraisals, which provokes UPFB as a way to cope with WFC/FWC. Previous studies on the stressor–emotion appraisal-behavior model of counterproductive work behavior have supported this conclusion (Miles et al., 2002; Rodell and Judge, 2009). Existing research has served to identify connections between WFC and threat appraisal (Glaser and Hecht, 2013; Zhao et al., 2019; Islam et al., 2020), WFC and anti-social work behavior (Morgan and King, 2012), and threat appraisal and unethical behavior (Jannat et al., 2021). However, this research is the first to state and test the relationship between WFC and UPFB *via* threat appraisal and UPFB.

Not all workers will react the same way to the similar daily conflict and cognitive appraisal. Lazarus’s research suggested that socio-cultural backgrounds help to explain why individuals’ responses and coping mechanisms could be different when they experience stress at work (Lazarus and Folkman, 1984). In this study, we focused on individual’s orientation toward family collectivism because it has been shown to be a cultural trait that is highly relevant to work-family issues (Masuda et al., 2019; Naqvi et al., 2022; Yang et al., 2022; Yuan et al., 2022). Family collectivism will influence the ability to cope with WFC/FWC. An orientation toward family collectivism may increase or decrease the possibility that workers will engage in UPFB.

**FIGURE 1:**
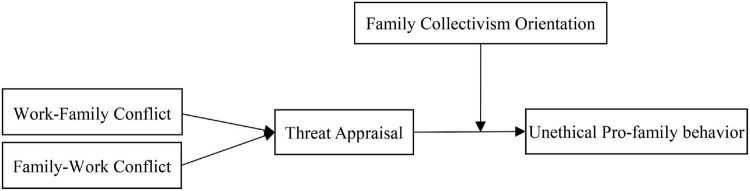
Hypothesized conceptual model of the research.

Overall, this research contributes to advance the ethics behavior literature in three significant aspects. First, this research extends the understanding of the antecedents of UPFB, which is small but ever increasing. WFC/FWC as distal antecedents and threat appraisal about such conflict as a proximal antecedent will induce employee UPFB. Second, this research addresses the gap in the literature regarding how WFC/FWC triggers UPFB. This research is the first to test the indirect path from WFC/FWC to UPFB *via* threat appraisal, drowning on cognitive appraisal theory, which permits an examination of how stressors and appraisals could predict the deployment of distinct coping mechanisms, particularly UPFB in the workplace. Third, this study considers a need to examine family collectivism orientation as a potential moderators of UPFB antecedents. This study develops hypotheses for the relationships that appear in Figure 1 and provide a more detailed discussion of these contributions in the following section.

## Theoretical Rationale and Hypotheses

### Cognitive Appraisal Theory

Cognitive appraisal refers to the process by which a person evaluates whether environmental cues relate to his or her wellbeing and, if so, how to cope with them (Lazarus and Folkman, 1984; Folkman et al., 1986). The cognitive appraisal process consists of two interrelated components: primary appraisal and secondary appraisal. In a primary appraisal, an individual estimates whether an external work stressor will be of harm or benefit to their work goals. When a primary appraisal indicates that the outcome of such a situation is irrelevant or beneficial to the individual, the appraisal process terminates. On the contrary, if the primary appraisal indicates that the situation could result in potential loss or harm to one’s wellbeing, the individual will conduct a secondary appraisal to determine what options are available to avert or minimize the loss or harm (Lazarus and Folkman, 1984).

Cognitive appraisal theory suggests that environmental cues and psychological characteristics could shape subsequent coping behaviors (Lazarus and Folkman, 1984; Gomes et al., 2013). Cognitive appraisal represents a critical mediator in the relationship between perceived stress and coping behavior (Lazarus and Folkman, 1984). Therefore, an individual’s perception of work-family conflicts, as well as their subsequent cognitive appraisal and coping behaviors, could be explained by the view of cognitive appraisal theory. Managers are often regarded as credible role models within the organization, and they are the most important prerequisites for driving the behavior of subordinate organizations. Therefore, leadership behavior is generally considered to be an important situational variable affecting employee behavior.

Since the development of cognitive appraisal theory in the 1980s, its fundamental principle has been empirically supported by numerous organizational behavior literature. For example, many studies have manifest how cognitive appraisal has served to explain work environments’ impacts on results, including work burnout (Glaser and Hecht, 2013; Simaes et al., 2021), behavioral disengagement (Cheng H. L. et al., 2021), counterproductive workplace behavior (Shoss et al., 2016), unethical behavior (Miao and Wang, 2017), and deviance behavior (Darrat et al., 2010). This study makes use of the literature on cognitive appraisal theory and extends it to include the work that has been conducted on UPFB. We suggest that UPFB on the part of the employee is one link of process from the stressor *via* cognitive appraisal to coping behavior. Concretely Speaking, we argue that WFC/FWC (stressor) is likely to induce threat appraisal (cognitive appraisal), which subsequently provokes employee UPFB (coping behavior). The strength of these relationships depends on the level of an employee’s orientation toward family collectivism.

### Work-Family Conflict/Family Work Conflict and Unethical Pro-family Behavior

Work and family conflicts are common and important sources of work stress in modern society. The conflict between work and family is a dilemma situation that the demands of work and family roles are in competition. When employees engage in pro-family unethical behavior, they not only need to convince themselves that their actions are justified, but they also need to believe that engaging in such behavior is not a risky proposition and that they can repay the family through their actions (Greenhaus and Beutell, 1985). In a word, family may interfere with work (FWC) and work may interfere with family (WFC) (Greenhaus and Beutell, 1985). These two conflicts both reflect a situation that people has insufficient individual resource (such as time and energy) or too much stress to fulfill the demands of their roles both at work and in the family at the same time (Glaser and Hecht, 2013). Because many researches on work and family conflicts have been conducted in the fields of organizational behavior and management psychology, work-related outcomes are core interests in that field (Amstad et al., 2011).

Although past studies have connected work-family interface conflicts with different kinds of work outcomes, there has been agreement that work-family interface conflicts impede desirable work outcomes (Miao and Wang, 2017), such as organizational citizenship behavior (Mercado and Dilchert, 2017), creativity (Babalola et al., 2021), job performance (Obrenovic et al., 2020), affective commitment (Tayfur Ekmekci et al., 2021), and job satisfaction (Netemeyer et al., 2005; Zhao et al., 2019). They also have had negative consequences, such as turnover intention (Post et al., 2009), job dissatisfaction (Islam et al., 2020), unethical behavior (Miao and Wang, 2017), anti-social behavior and counterproductive behavior (Morgan and King, 2012; Mercado and Dilchert, 2017). That is to say, studies have always depicted the consequences of work and family interface conflict in a negative light. UPFB is a kind of negative actions in the organization (Liu et al., 2020). Here, we expect that work-family conflict will lead to UPFB.

Unethical pro-family Behavior often has occurred for the wellbeing of the family (Liu et al., 2020). Employees who have experienced work and family conflicts have been challenged to fulfill their family responsibilities (Glaser and Hecht, 2013). Work-family conflict and family work conflict decreased wellbeing of the family because such conflicts put employees in a situation where they have been unable or have to consume a lot of resources to balance work and family (Obrenovic et al., 2020). Employees were inclined to engage in UPFB as a means by which to strike a better balance between the two. Consequently, their actions could reflect a means by which to compensate the family. For example, taking company property home for the family to enjoy could represent a type of compensation to the family (Liu et al., 2020). When confronted with family work conflicts, employees have been more likely to engage in UPFB—such as using work resources to deal with family affairs—so that they could extricate themselves from the conflict and return to a state of balance in a timely fashion. When faced frequently with work-family conflicts or family work conflicts, an employee’s motivation to engage in UPFB could increase. Therefore, work-family conflict and family work conflict may increase UPFB in the worksite.

**H1:** Work-to-family conflicts (H1a) and family to-work conflicts (H1b) are positively related to UPFB.

### Mediating Effect of Threat Appraisal

Cognitive appraisal theory has suggested that the response to a stressor, such as work-family conflict or family work conflict, depended on an appraisal of the stressor (Lazarus and Folkman, 1984). Previous studies have suggested that cognitive appraisal is an important predictor of coping behavior (Folkman et al., 1986). Hence, we predicted that the relationship between WFC/FWC and UPFB would be mediated by threat appraisal. When faced with WFC or FWC, an employee might first conduct an appraisal. They might perceive that the problems experienced were due to an inability to meet the role demands of work or family domain. Consequently, they might conduct a secondary appraisal to weigh their options with regard to coping and corresponding courses of action.

Work-family conflict or FWC has been defined as a role conflict; crossover role requirements interfere with the performance of the tasks associated with the roles played (Greenhaus and Beutell, 1985). When employees face the conflict between work and family, they may not be able to balance the demands of the two roles. The imbalance identified could significantly compromise their ability to fulfill their responsibilities at work or in their family (O’Connor et al., 2010). Such role stress would typically prompt employee to evoke a negative response (Simaes et al., 2021). Studies have shown that both WFC and FWC have been associated with unsatisfied (Zhang et al., 2019), emotional exhaustion (Glaser and Hecht, 2013), and burnout (Simaes et al., 2021), lower job performance as well as stunted career development (Hoobler et al., 2010). These negative outcomes may lead employees to doubt about their self-worth and ultimately also impact self-esteem (Glaser and Hecht, 2013). Given that, work and family conflicts frequently could be perceived as a potential threat.

In order to cope with the threatening situation of role conflicts, employees may engage in UPFB. Some studies have documented the ways that unethical behavior has been positively related to the perceived threat (Kouchaki and Desai, 2015; Jannat et al., 2021). Threat appraisal has been shown to influence unethical behavior significantly (Jannat et al., 2021). Several recent studies have also tested the mediating role of threat appraisal between WFC/FWC and work outcomes (Glaser and Hecht, 2013; Zhao et al., 2019; Islam et al., 2020). Therefore, we also argue that the positive relationship between WFC/FWC and UPFB is mediated by the appraisal of threat. We propose the following hypothesis:

**H2:** Employees’ threat appraisal mediates the relationships between Work-to-family conflicts (H1a) and family to-work conflicts (H1b) and UPFB.

### Moderating Effect of Family Collectivism

Lazarus emphasized that the socio-cultural context played an important role in cognitive appraisal (Lazarus and Folkman, 1984). Different social-cultural contexts would result in the expression of different coping mechanisms and emotional responses to stress (Lazarus and Folkman, 1984; Singelis et al., 1995). The most salient source of cross-cultural variation has proven to be the relationship between individualism and collectivism (Hofstede, 2006; Heine and Ruby, 2010). Every individual possesses both individualistic and collectivism orientations, but the degree to which either one is expressed would depend on their cognitive systems (Triandis, 1995). Collectivism implies that people’s actions are motivated by collective goals and group norms (Kemmelmeier et al., 2003). Individuals with collectivism orientation are more likely to construct strong social-emotional connections with other members of the group or family (Cross et al., 2000). They would be more likely to put the interests of the group (or family) ahead of their individual interests (Singelis et al., 1995; Triandis, 1995). The individualism-collectivism spectrum collected in the GLOBE data was based primarily on issues about family rather than organization; collectivism was linked to cohesiveness and loyalty within the family (Hofstede, 2006). Therefore, family collectivism can be regarded as a dimension of collectivism.

Previous studies have shown that family collectivism has been relevant to work-family issues (Zhang et al., 2019). Considering that, this study focused on family collectivism (House et al., 2004). Family collectivism has been defined as a value orientation to which individuals prioritize their family interests (House et al., 2004; Zhang et al., 2019). The level of family collectivism could vary significantly among individuals (Zhang et al., 2014).

People devoted to family collectivism have demonstrated high levels of identification with their families (Hofstede, 2006). Consequently, they put an interest in their families over individual interests. People who have invested a good amount of time or effort into their work roles also could orientate toward family collectivism if they believed that their hard work would benefit the family (Zhang et al., 2019). Family collectivism has been characterized as an ideology that encourages people to treat hard work as a necessary method that yields monetary resources to benefit their family (Yang et al., 2000; Zhang et al., 2019). Studies have demonstrated that these people adhere to these beliefs as they develop their roles at work and in the family (Yang et al., 2000; Zhang et al., 2014, 2019). Ollier-Malaterre and Foucreault (2017) also noted that individuals in collectivism cultures reported less work-family conflict than individuals in individualistic cultures. When people who value family collectivism conduct an appraisal of the work-family conflicts that they experienced, they may not perceive threats because the fulfillment of their work roles benefited their families (Zhang et al., 2019). Therefore, a person’s high levels of family collectivism are not necessarily related to an appraisal of greater threat from WFC. However, once a threat appraisal has been performed, people with greater orientation toward family collectivism tended to have a stronger motivation to engage in UPFB; they believed that the purpose of their actions would be to contribute to the welfare of their families (Tatliyer and Gur, 2021). Individuals will feel guilty and ashamed emotionally after engaging in unethical behaviors in the family, and subsequent unethical behaviors in the family due to guilt and shame are inhibited. Therefore, we suggest that the relationship between threat appraisal and UPFB is moderated by family collectivism orientation. We propose the following:

**H3:** The positive relationship between threat appraisal and UPFB is moderated by Family collectivism orientation. This positive relationship is stronger among people with higher (rather than lower) levels of family collectivism orientation.

**H4:** The mediating effect of threat appraisal is stronger among people with higher level (rather than lower level) of orientation toward family collectivism.

## Materials and Methods

### Data Collection and Sample

There are two main forms of measuring the unethical behavior of pro-family, one is the questionnaire method and the other is the experimental method. The participants for this research were recruited from the alumni of two universities in Northern China. The participants resided in multiple regions and worked in a variety of professions. With the help of the school’s alumni administration office, we posted a QR code of the questionnaire to alumni virtual chat groups (a kind of online community) *via* WeChat and QQ, an instant communication App that is widely used in China. A letter of introduction was attached to the questionnaire that explained why we constructed this survey, assured participants that the data collected would remain completely confidential and notified the participants that they could quit the survey at any time.

The surveys were conducted online and in Chinese. The participants completed two surveys at two points in time with a 1-month lag between them. We received 1130 alumni completed questionnaires during the first stage and 496 completed questionnaires during the second stage. The total response rate was 43.89% after removing the invalid questionnaires. A total of 52.8% of the respondents were female, and 72.8% of the respondents were married. Most of the respondents had an undergraduate degree (77.9%). More than a third (32.3%) of the participants had held their current job for less than 3 years. A little more than a quarter of them had been at their current job from 4 to 6 years (28.6%). Approximately one-fifth of the participants (21.4%) had their jobs for 7 to 10 years. Only 17.7% of the participants had worked at their current job for over 10 years.

### Measures

We translated and conducted a back translation of all of the scales (Chinese-English) (Behr, 2017). The items on the questionnaire were rated on a five-point Likert scale ranging from 1 (strongly disagree) to 5 (strongly agree).

#### Work-Family Conflict and Family Work Conflict

During the first stage of the study, participants reported the level of perceived WFC and FWC over the past month using Netemeyer et al. (1996)’s 10-item scale (Netemeyer et al., 1996). The WFC and FWC scales each included five items. Higher scores indicated greater levels of conflict. Sample items from the questionnaire on WFC included “Because of my job responsibilities, I have to change the plan of my family activities” and “Because of work pressure, I find it hard to fulfill my family obligations.” Sample items of FWC included “Because of family pressures, affect my ability to perform my job duties, I find it hard to perform work-related duty well” and “Because of demands on my time at home, I have to Put off or give up some tasks at work.” The Cronbach’s alpha of WFC and FWC were 0.820 and 0.824, respectively.

#### Threat Appraisal

During the first stage of the study, participants provided ratings of their threat-appraisal using an adapted version of Glaser and Hecht’s six-item scale (Glaser and Hecht, 2013). The stem for each question was “if my family and my work interfere with each other, I would feel that …” An example of responses included, “I may lose the affection of someone important to me.” The Cronbach alpha for the scale was 0.811.

#### Family Collectivism Orientation

During stage 2 of the study, participants provided ratings of their family collectivism orientation. The scale included three items adapted from Zhang et al.’s (2019) work, which was first developed by Triandis (1996). A sample item was “I prioritize my family welfare to myself interest or goal.” The Cronbach alpha was 0.765.

#### Unethical Pro-family Behavior

During the second stage, participants also reported the frequency of their UPFB over the past month. The scale included seven items developed by Liu et al. (2020). A sample item was “I use work resources to help to deal with issues related to my family at work.” The Cronbach alpha was 0.902.

#### Control Variables

Previous studies on work and family relationships have suggested that demographic variables may be associated with UPFB. In order to reduce the biases caused by demographic factors, this research used these control variables: Age, gender, marital status, education, and organizational tenure.

### Analytical Strategy

#### Measurement Model

Prior to hypotheses testing, we tested our measurement model through confirmatory factor analyses using Mplus 7. Confirmatory factor analyses showed that the five-factor model fitted the data well: X^2^/df = 2.367, CFI = 0.937, TLI = 0.929, SRMR = 0.050. The results appear in Table 1.

**TABLE 1:** Results of confirmatory factor analyses.

Model	X^2^	df	X^2^/df	CFI	TLI	SRMR
Five-factor model: WFC, FWC, TA, FCO, UPFB	684.187	289	2.367	0.937	0.929	0.050
Four-factor model: WFC + FWC, TA, FCO, UPFB	829.527	293	2.831	0.914	0.905	0.056
Three-factor model: WFC + FWC, TA + FCO, UPFB	1122.531	296	3.792	0.868	0.855	0.065
Two-factor model: WFC + FWC + TA, FCO + UPFB	1475.712	298	4.952	0.811	0.794	0.074
One-factor model: WFC + FWC + TA + FCO + UPFB	1733.131	299	5.796	0.770	0.750	0.076

*WFC, work-family conflict; FWC, family work conflict; TA, threat appraisal; FCO, family collectivism orientation; UPFB, unethical pro-family behavior.*

#### Descriptive Statistics Hypothesis Testing

We used SPSS 22 to analyze the means, standard deviations, and inter-correlations for the variables. The results appear in Table 2.

**TABLE 2:** Means, standard deviations, and correlation coefficients of variables.

	Mean	*SD*	1	2	3	4	5	6	7	8
1. Gender	1.530	0.500								
2. Marital status	1.720	0.450	0.023							
3. Education	1.990	0.760	−0.090	0.001						
4. Tenure	3.120	10.274	0.069	0.322**	0.001					
5. Work-family conflict	3.390	0.667	0.046	0.139**	0.011	0.072				
6. Family work conflict	3.352	0.690	0.044	0.201**	0.021	0.007	0.663			
7. Threat appraisal	3.611	0.667	0.017	0.297**	0.071	0.036	0.427**	0.513**		
8. Family collectivism orientation	3.440	0.903	0.016	0.044	0.003	−0.145**	0.561**	0.518**	0.342**	
9. Unethical pro-family behavior	3.416	0.841	0.053	0.126**	0.025	0.022	0.571**	0.620**	0.649**	0.492**

N = 496. *p < 0.05; **p < 0.01.

#### Hypothesis Testing

This moderated mediation hypothesis model was tested through the method and PROCESS macro that developed by Preacher and Hayes research (Preacher et al., 2007; Hayes, 2013). First, we tested Hypotheses 1 and 2. We analyzed simple mediation using the Model 4 of the PROCESS macro embedded in SPSS; the findings are presented in Table 3. Results showed that work-family conflict was significantly and positively related to UPFB (*B* = 0.360, *SE* = 0.058, *p* < 0.001), supporting Hypothesis 1a. Family work conflict was significantly and positively related to UPFB (*B* = 0.524, *SE* = 0.056, *p* < 0.001), supporting Hypothesis 1b. We then tested the indirect effect of work-family conflict on UPFB *via* threat appraisal (H2a), and found that the indirect effect was statistically significant (*B* = 0.253, *SE* = 0.041, 95% CI [0.174, 0.337]), supporting Hypothesis 2a. In the same way, the indirect effect of family work conflict on UPFB *via* threat appraisal also was statistically significant (*B* = 0.272, *SE* = 0.038, 95% CI [0.197, 0.348]), supporting Hypothesis 2b.

**TABLE 3:** Regression results of mediating and moderating effects.

Outcome variable: Unethical pro-family behavior	*B*	*SE*	t	*R* ^2^
				0.432
Constant	0.166	0.197	0.845	
Gender	0.041	0.058	0.713	
Marital status	0.044	0.038	1.154*	
Education	−0.001	0.069	−0.008	
Tenure	0.002	0.024	0.090	
Work-family conflict	0.360	0.058	6.240***	
Family work conflict	0.524	0.056	9.341***	
Mediator: Threat appraisal	Effect	*SE*	Boot LL 95% CI	Boot UL 95% CI
Indirect effect of Work-family conflict on Unethical pro-family behavior	0.253	0.041	0.174	0.337
Indirect effect of Family work conflict on Unethical pro-family behavior	0.272	0.038	0.197	0.348
				0.525
Constant	1.019	0.350	2.912***	
Gender	0.070	0.053	1.316	
Marital status	0.075	0.066	1.140	
Education	0.018	0.035	0.513	
Tenure	0.038	0.022	1.701	
Threat appraisal	0.247	0.122	2.024*	
Family collectivism orientation	0.141	0.110	1.282*	
Threat appraisal*Family collectivism orientation	0.158	0.039	4.039**	

Presented results are unstandardized effects. N = 496. *p < 0.05; **p < 0.01; ***p < 0.001. Bootstrap sample size = 5,000.

Second, we tested Hypothesis 3. We analyzed simple moderation (Hypothesis 3) using Model 1 of the PROCESS macro embedded in SPSS; the results appear in Table 3. As expected, we found a significant interactive effect of threat appraisal and family collectivism orientation on UPFB (*B* = 0.158, *SE* = 0.039, *p* < 0.01), supporting Hypothesis 3.

**FIGURE 2:**
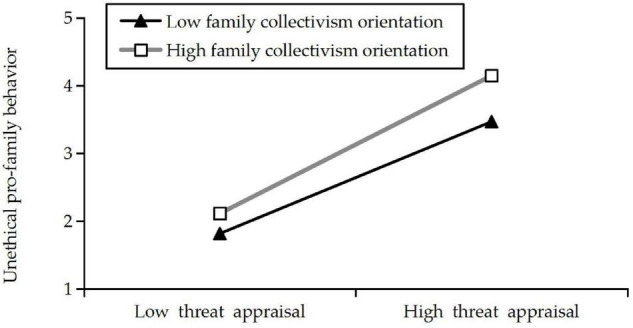
Interactive effect of threat appraisal and family collectivism orientation on unethical pro-family behavior (UPFB).

Then, we conducted a simple slope test to verify whether the effects were in the predicted direction based on Aiken and West’s (1991) work. The result revealed that the relationship between threat appraisal and UPFB was positive and significant at the low level (−1 *SD*) of family collectivism orientation (simple slope = 0.49, *SE* = 0.070, *p* < 0.001). When the level of family collectivism orientation was high (+ 1 *SD*), the relationship between threat appraisal and UPFB was still remained positive and significant, but the slope was stronger (simple slope = 0.77, *SE* = 0.048, *p* < 0.001). This interaction is shown in Figure 2.

Finally, we tested Hypothesis 4. We used an overall moderated-mediation model, Model 14 of the PROCESS macro embedded in SPSS. The data of the continuous variables were mean-centered before examining the hypotheses, which was suggested by Aiken and West (1991). The results appear in Table 4. Bootstrapping analyses showed that when family collectivism orientation was at low level (−1 *SD*), the conditional indirect effect of work-family conflict on UPFB *via* threat appraisal was statistically significant (*B* = 0.161, *SE* = 0.045, 95% CI [0.075, 0.253]). Moreover, when family collectivism orientation was at high level (+ 1 *SD*), the conditional indirect effect also was significant (*B* = 0.265, *SE* = 0.043, 95% CI [0.182, 0.351]). Furthermore, we found that when family collectivism orientation was low (−1 *SD*), the conditional indirect effect of family work conflict on UPFB *via* threat appraisal was statistically significant (*B* = 0.215, *SE* = 0.049, 95% CI [0.114, 0.306]). When family collectivism orientation was high (+ 1 *SD*), the conditional indirect effect was still remained significant (*B* = 0.277, *SE* = 0.042, 95% CI [0.195, 0.360]. Hypothesis 4a and 4b were thus supported.

**TABLE 4:** Moderated-mediation results at different levels of family collectivism orientation.

Indirect effect	Level of family collectivism orientation	Boot indirect effect	Boot *SE*	Boot LL 95% CI	Boot UL 95% CI
Work-family conflict→Threat	−1 *SD*	0.161	0.045	0.075	0.253
Appraisal→Unethical pro-family	M	0.213	0.041	0.135	0.297
Behavior	+ 1 *SD*	0.265	0.043	0.182	0.351
Family work conflict→Threat	−1 *SD*	0.215	0.049	0.114	0.306
Appraisal→Unethical pro-family	M	0.246	0.041	0.165	0.326
Behavior	+ 1 *SD*	0.277	0.042	0.195	0.360

*Presented results are unstandardized effects. N = 496. Bootstrap sample size = 5,000.*

## Discussion

Drawing on cognitive appraisal theory, this research develops and tests a model of the antecedents of UPFB in the workplace. The result of the research keeps in touch with the hypothesis model as predicted. Specifically, the findings demonstrated that WFC and FWC encouraged a threat appraisal, which was related to employees’ UPFB. The impact of WFC and FWC on UPFB partly resulted from a threat appraisal. This study also examined family collectivism orientation as a moderator of the mediational path between threat appraisal and UPFB. The findings revealed that family collectivism orientation strengthened the relationship between threat appraisal and UPFB. The findings also revealed that family collectivism orientation conditionally moderated the indirect effect of WFC and FWC on UPFB (*via* threat appraisal). Hence, this study confirmed that various levels of family collectivism orientation could capture differences in reactions to certain types of work stress and threat appraisals.

### Theoretical Implications

There are several important theoretical implications of this research. First, UPFB, as a unique form of unethical behavior in the workplace, only recently has received scholarly attention (Liu et al., 2020; Cheng K. et al., 2021; Yao et al., 2021). Therefore, understandings about effective ways to reduce employees’ UPFB is limited. The causes of UPFB have not been adequately identified. In response to the call for more research on the antecedents of UPFB (Liu et al., 2020), we began with WFC and FWC because both have been shown to induce negative outcomes (Morgan and King, 2012; Mercado and Dilchert, 2017; Miao and Wang, 2017). The effective way to reduce UPFB is to address its causes (Cheng K. et al., 2021). Verification of the positive effects of WFC and FWC on UPFB represents an important contribution to the limited research on the causes of UPFB. Viewed from another perspective, this research also enriches the work on the work-family interface by extending the scope of outcomes of WFC and FWC to include family related unethical behavior in the organization. This extension is small but important because previous studies didn’t adequately capture the importance of family related stress.

This empirical study advances the emerging research on UPFB by exploring proximal (threat appraisal) and distal (WFC and FWC) antecedents. The study explicit proposal and empirical examination of employees’ threat appraisals as a mechanism by which WFC and FWC relate to UPFB, led to a possible explanation of why employees who experienced WFC and FWC were possibly to engage in UPFB. The mediating role of threat appraisal identified in this study showed that the impact of WFC and FWC on UPFB could be understood as a process of cognitive appraisal. This finding deepens current understandings of UPFB and its causes; they could be coping mechanisms for stress. These findings are in accordance with the theory of adaptation to stress (Lazarus and Folkman, 1984), which suggest that stress coping behavior itself is not directly determined by the stressor itself, but though the mediator of one’s cognitive appraisal. Organizational identity, perceived obligation, and family based self-esteem all have unique mechanisms to influence pro-family unethical behavior, but co-existing psychological factors may have a synergistic effect on pro-family unethical behavior, so the interaction of these three psychological factors Effects may have different effects on pro-family unethical behavior.

Finally, this study also makes a contribute to the literature through examining a moderator as antecedents of UPFB. Specifically, we found that family collectivism orientation influenced the threat appraisal-UPFB relationship and the mediation path from WFC and FWC to UPFB *via* threat appraisal.

### Practical Implications

The general consensus is that UPFB is destructive to organization and its members due to its costly consequences in the workplace. Hence, it is important for organizations and their managers to understand what triggers employees to engage in such behavior. The findings of the current study provide important insights through the identification of employee threat appraisal and WFC/FWC as possible factors that lead to UPFB. In order to reduce the incidence of employee UPFB, managers must carefully observe the interface of employees’ work and family relationships and cautiously monitor the negative appraisals that could occur. There are currently three cognitive mechanisms, namely moral neutralization, moral evasion, and moral neglect.

This study’s findings suggest that organizations and their managers should actively seek to reduce employees’ WFC and FWC. Management should promote a balance between employees’ work and family roles when jobs are designed and workplace policies established. They could develop policies that include work-family support—such as flexible work hours and childcare—because they would be conducive to the organization’s long-term development. Employees who struggle with WFC or FWC could be encouraged to consider seeking support from the organization. These steps could help to prevent UPFB.

Sometimes conflicts between work and family are unavoidable. Employees could benefit from assurances that they do not have to represent an ongoing threat. Therefore, managers should train employees on cognitive appraisal because it could help them understand the relationship between work and family in a more positive way. Given the impact of this self-control, organizations should shape an ethical organizational culture, such as creating an ethical organizational climate by recognizing good people and deeds.

It is commendable that people with family collectivism orientation wish to take responsibility for the wellbeing of their families. However, UPFB violates societal and organizational norms. UPFB has a negative impact on the organization, but also affects the career development of employees. Employees’ families also could ultimately suffer negative consequences. Managers should strengthen the training of employees’ collectivism orientation, help employees establish a positive family collectivism orientation, and encourage employees to reflect on what kind of work mode is truly of long-term benefit to the family.

### Limitations and Future Directions of Research

However, we have to acknowledge that this research has several limitations to merit attention. First, the focus solely on Chinese employees could limit the relevance of the findings to other countries. Therefore, the future research could test the hypothesis model in other settings.

Second, the reliance on self-reporting could have yielded responses that hide some of the participants’ actual actions. However, the cognitive appraisal information collected would have been difficult to obtain from any other source. Additionally, it would have been difficult to learn of UPFB in an accurate way from other sources because they are actions that are hidden.

Third, it was difficult to adequately determine causality, despite the fact that data was collected at two different times. The variables were correlational but not necessarily causal. To adequately address this limitation, future research could be constructed by various research designs (e.g., longitudinal study, quasi-experimental and experimental) to provide convincing evidence of any causal relationships.

Fourth, there could be other potential mediators of the relationships studied. This work was grounded in cognitive appraisal theory. Future research could include additional potential mediators adopted from other theoretical frameworks, such as resource conservation theory. There could be another explanation for the hypothesized relationships that could also be informed by a different conceptual framework. For example, Hobfoll’s theory of resource conservation shows that the situations of external need are possible to consume personal resources, leading to stress as well as attitudinal responses or negative behavior to stress (Hobfoll, 1989). Emotional exhaustion may be a variable that could transfer the impact of WFC/FWC on UPFB. Previous studies have demonstrated the positive effect of WFC/FWC on emotional exhaustion (Glaser and Hecht, 2013) as well as the positive relationship between emotion exhaustion and unethical behavior (Qi et al., 2020).

Finally, family collectivism was only used as a moderator in the second stage of this study. Future studies could explore other possible moderators (e.g., self-efficacy) in the first or/and second stage. Previous studies have shown that self-efficacy may make individuals resilient to the negative influence of work-family conflicts (Glaser and Hecht, 2013; Hao et al., 2015). A promising direction for future research would test the moderating role of self-efficacy and the ways that it may affect individuals’ perceptions of their abilities to manage various conflicts and determine their coping strategies.

This paper has data limitations, i.e., a study was originally designed, but data limitations prevented it from being carried out; methodological limitations, i.e., certain methods have not yet been broken through and become the direction to be attacked in future studies; theoretical limitations, i.e., its own theoretical foundation is not yet perfect, and the analysis and summary of conclusions are limited.

## Data Availability Statement

The original contributions presented in this study are included in the article/supplementary material, further inquiries can be directed to the corresponding author.

## Author Contributions

ML, LZ, ZZ, and XH were responsible for designing the framework of the entire manuscript from topic selection to solve to experimental verification. All authors contributed to the article and approved the submitted version.

## Conflict of Interest

The authors declare that the research was conducted in the absence of any commercial or financial relationships that could be construed as a potential conflict of interest.

## Publisher’s Note

All claims expressed in this article are solely those of the authors and do not necessarily represent those of their affiliated organizations, or those of the publisher, the editors and the reviewers. Any product that may be evaluated in this article, or claim that may be made by its manufacturer, is not guaranteed or endorsed by the publisher.
